# Integrating habitat suitability, phytochemical quality, and landscape stability to identify conservation priorities for *Arnebia guttata* Bunge under climate change in East Asia

**DOI:** 10.3389/fpls.2026.1890224

**Published:** 2026-08-06

**Authors:** Yaqiong Bi, Feng Gao, Chunhong Zhang, Guoqing Han, Chimedragchaa Chimedtseren, Myadagbadam Urtnasan, Zeyuan Zhao, Feibiao Shan, Wenle Wang, Xuhong Duan, Minhui Li

**Affiliations:** 1 Inner Mongolia Traditional Chinese & Mongolian Medical Research Institute, Hohhot, China; 2 Inner Mongolia Medical University, Hohhot, China; 3 Baotou Medical College, Baotou, China; 4 Institute of Traditional Medicine and Technology of Mongolia, Ulaanbaatar, Mongolia; 5 College of Pharmacy, Hebei University of Chinese Medicine, Shijiazhuang, China

**Keywords:** *Arnebia guttata* Bunge, East Asia, ecological niche overlap, landscape fragmentation, phytochemical quality, species distribution models

## Abstract

*Arnebia guttata* Bunge is a threatened medicinal plant predominantly distributed in arid and semi-arid regions of China and Mongolia. Increasing medicinal demand and ongoing climate change highlight the need for an integrated evaluation of its habitat dynamics, medicinal quality, and ecological stability. In this study, we combined species distribution modeling, niche analysis, phytochemical evaluation, and landscape fragmentation analysis to assess habitat suitability, medicinal quality, and habitat stability within a unified framework. Results showed that the suitable habitats of *A. guttata* are projected to remain relatively stable while moderately expanding toward the Altai, Qilian, Helan, and Tianshan Mountains by the end of the 21st century. Hydrological variables exerted dominant control over habitat suitability, whereas medicinal quality showed comparatively weaker and more spatially heterogeneous relationships with environmental conditions. Areas with relatively high phytochemical quality were more spatially restricted and environmentally differentiated than climatically suitable habitats. In addition, suitable habitats and phytochemical quality zones differed markedly in spatial distribution and environmental characteristics, suggesting that ecological suitability does not necessarily correspond to medicinal quality. Landscape analysis further revealed clear differences in fragmentation and connectivity among habitat types.

## 1 Introduction

Medicinal plant resources represent a unique and complex system, and their conservation and sustainable utilization are particularly critical for endangered and vulnerable species. *Arnebia guttata* Bunge is a perennial herb belonging to the Boraginaceae family and is widely used in traditional medicine in China and Mongolia, possessing high medicinal and industrial economic value. The dried root of *A. guttata*, known as Arnebiae Radix, has been used for centuries traditional Chinese medicine and Mongolian medicine. It is widely applied in the treatment of inflammatory and skin-related disorders and also serves as an important natural pigment source in the pharmaceutical, cosmetic, textile, and food industries (Park et al., 2020; Ma et al., 2021; Chinese Pharmacopoeia Commission, 2025; Bao et al., 2025).

From a phytochemical perspective, *A. guttata* accumulates diverse bioactive constituents, among which naphthoquinones are regarded as the principal pharmacologically active compounds (Park et al., 2018; Zhang et al., 2018; Wang et al., 2020; Feng et al., 2020; Rat et al., 2023). The spatial variation of these metabolites is closely associated with environmental conditions and may influence the medicinal quality of natural populations.

*A. guttata* is distributed across arid and semi-arid regions of East Asia, particularly in northern China and Mongolia. It has been evaluated as ‘Vulnerable’ in the Chinese Red List of Biodiversity due to fragmentation, limited distribution, and anthropogenic disturbances. Natural populations are typically small, isolated, and occur in fragile environments such as deserts and rocky slopes, making them highly sensitive to environmental changes. Additionally, the species still relies predominantly on wild harvesting because standardized cultivation systems remain underdeveloped, further increasing pressure on natural populations (Wang et al., 2025b, Liu et al., 2024a).

Medicinal plants are integral components of natural ecosystems and are highly sensitive to climate change and anthropogenic disturbance (Laurance, 2019; Keys et al., 2019; Theodoridis et al., 2023). Climate warming is expected to drive distribution shifts toward higher latitudes and elevations, while land-use change and overexploitation further intensify habitat fragmentation and ecological degradation (Rana et al., 2022; Manish, 2022; Wang et al., 2025a). These interacting pressures may jointly affect habitat suitability, population persistence, and medicinal resource sustainability, particularly for vulnerable species with narrow ecological tolerances (Chen et al., 2016; Ci et al., 2023).

Species distribution models (SDMs) have become important tools for predicting species distributions and assessing environmental suitability (Elith and Leathwick, 2009; Hao et al., 2019). Ensemble modeling frameworks, such as Biomod2, integrate multiple algorithms to improve predictive performance and reduce model uncertainty (Araújo and New, 2007; Thuiller et al., 2009). By quantifying climatic niches and potential distribution shifts, SDMs help characterize species vulnerability, adaptive potential, and conservation prioritization (Guisan and Thuiller, 2005; Guisan et al., 2014; Liu et al., 2024a). However, previous studies have largely treated habitat suitability, medicinal quality, and ecological stability as equivalent concepts, although these aspects may not overlap spatially or respond to the same environmental conditions. Most studies have also relied primarily on climatic variables or single-model approaches, with limited consideration of phytochemical quality, habitat fragmentation, and multi-factor interactions across large spatial scales. To address the potential mismatch between ecological suitability, medicinal quality, and long-term habitat stability, we defined three hierarchical habitat categories: core stable suitable habitats, phytochemical quality advantage zones, and high-quality stable habitats. This framework enables comparative evaluation of habitat suitability, medicinal quality, and long-term habitat stability allowing direct comparison among habitat suitability, medicinal quality, and habitat stability.

This study integrates species distribution modeling, niche analysis, phytochemical assessment, and landscape pattern analysis to evaluate the future habitat dynamics and medicinal quality of *A. guttata* under climate change. Specifically, we aimed to: (1) characterize the current and future spatial patterns of habitat suitability for *A. guttata* under climate change; (2) evaluate niche dynamics and distributional stability across future climate scenarios; (3) identify the environmental drivers associated with habitat suitability and medicinal phytochemical quality; and (4) compare the spatial mismatch, environmental differentiation, and landscape fragmentation among core stable suitable habitats, phytochemical quality advantage zones, and high-quality stable habitats. Our results further show that climatically suitable habitats do not necessarily coincide with areas of high medicinal quality, emphasizing the need to jointly consider ecological suitability, medicinal quality, and habitat stability in conservation planning.

## 2 Materials and methods

### 2.1 Sample information

#### 2.1.1 Occurrence records of *A. guttata*

**Figure 1:**
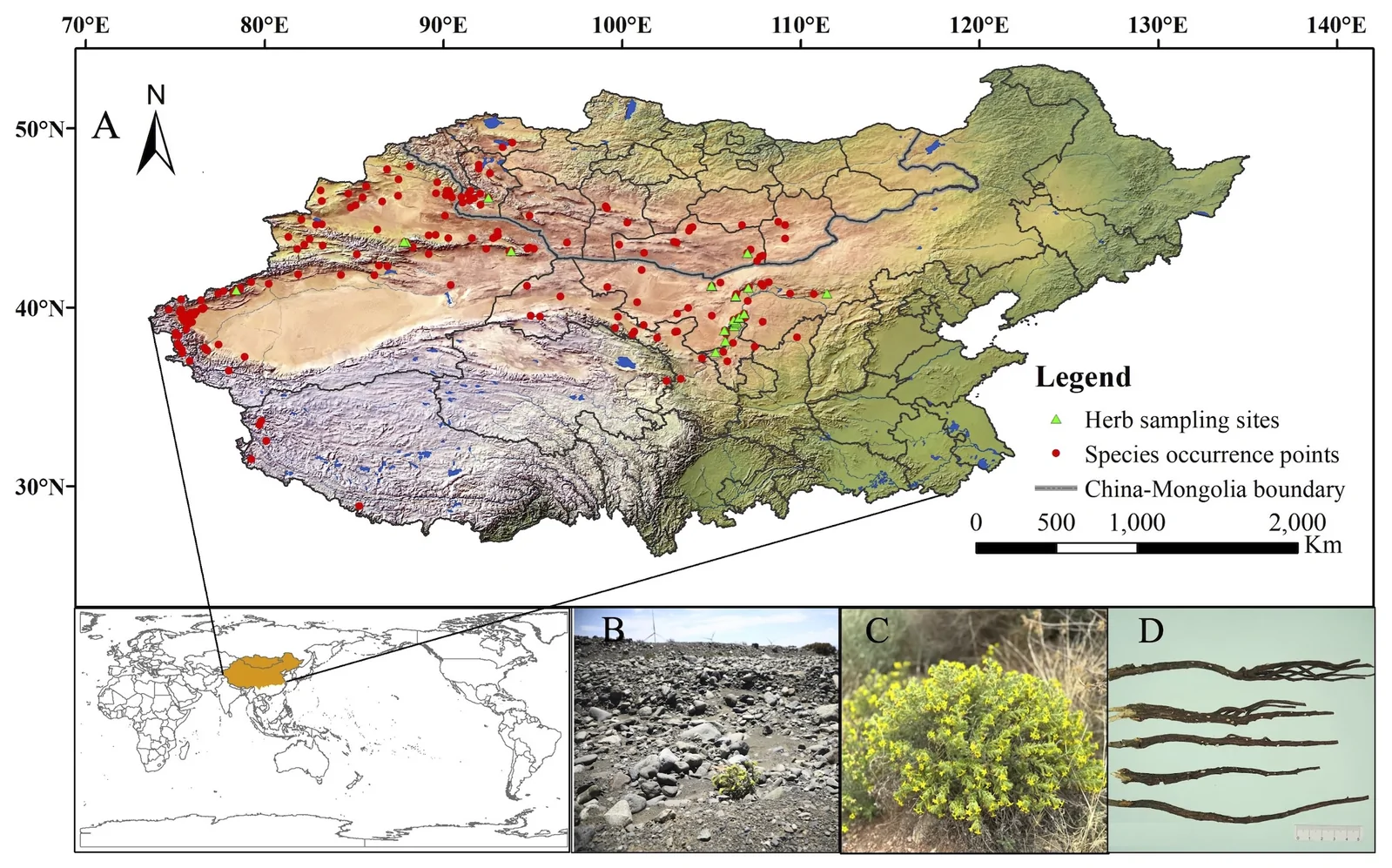
Sampling locations and morphological features of *A. guttata*. **(A)** Topographic map showing the sampling sites of the species; **(B)** Plant habitat; **(C)** Perennial plant; **(D)** Perennial root.

In this study, occurrence records of *A. guttata* were compiled from multiple sources, including the Global Biodiversity Information Facility (GBIF, https://www.gbif.org/), the Chinese Virtual Herbarium (CVH, https://www.cvh.ac.cn/), and the National Specimen Information Infrastructure (NSII, http://www.nsii.org.cn/2017/home.php). Only records collected after 1990 were retained to ensure consistency with the current climate dataset, resulting 281 occurrence records used for subsequent analyses. In addition, field surveys on traditional medicinal plant resources were conducted by our research group between 2012 and 2024 in China and Mongolia, providing supplementary distribution records for *A. guttata*. ArcGIS 10.8 was employed to remove occurrence records falling outside the geographic boundaries of East Asia. All occurrence records were subjected to rigorous data cleaning procedures. Records lacking geographic coordinates or with obvious spatial errors were removed. Duplicate records within the same grid cell were excluded to avoid spatial sampling bias. To minimize spatial sampling bias and reduce the likelihood of model overfitting caused by clustered records, spatial thinning was applied using the R package spThin (Aiello-Lammens et al., 2015). In this procedure, only a single occurrence point per species was retained within each 5 km grid cell, thereby improving the robustness of subsequent analyses (Boria et al., 2014). After filtering, a total of 187 valid occurrence records were retained for *A. guttata* (**Figure 1**).

#### 2.1.2 Medicinal plant sampling of *A. guttata*

To support the evaluation of medicinal material quality, 25 plant samples were collected from representative natural populations across the species’ main distribution range between 2023 and 2025, covering Inner Mongolia, Ningxia, Xinjiang, and Mongolia (**Figure 1**). Medicinal plant samples were primarily gathered from the eastern part of the species’ distribution, particularly Inner Mongolia and Ningxia, where natural populations are relatively clustered and easily accessible. The collected samples were used for subsequent phytochemical analyses of major bioactive compounds and for quality suitability modeling.

### 2.2 Environmental variables and geospatial data

In total, 43 environmental predictors were assembled for analysis, including climate, soil, topography, and anthropogenic factors (**Supplementary Table 1**). Climatic variables were derived from the WorldClim database (https://www.worldclim.org/), based on simulations of the BCC-CSM2-MR model under the CMIP6 framework of the Intergovernmental Panel on Climate Change (IPCC). The BCC-CSM2-MR model has demonstrated improved capability in reproducing key climate variables, including temperature and precipitation, at both global and regional scales, with notable performance in East Asia (Wu et al., 2019). Given its validated regional performance, it was selected for climate projection in this study. However, it should be noted that the use of a single general circulation model may introduce uncertainty in future climate projections, particularly in arid and semi-arid regions such as Mongolia, where precipitation variability is high. Therefore, results derived from future climate scenarios should be interpreted with caution. Nineteen bioclimatic variables (BIO1-BIO19) were obtained for the baseline period (1970-2000) and for future scenarios averages over 20-year periods (2021-2040, 2040-2060, 2061-2080, 2081-2100) under four Shared Socio-economic Pathways (SSPs): 126, 245, 370 and 585. These scenarios represent contrasting future socio-economic trajectories and policy environments, spanning a broad range of greenhouse gas emission pathways. Specifically, SSP126 represents a sustainability-oriented pathway with strong mitigation efforts; SSP245 corresponds to a “middle-of-the-road” socio-economic development pathway; SSP370 reflects a medium-to-high emission reference scenario associated with regional rivalry and limited international cooperation; and SSP585 represents a high fossil-fuel development world with minimal mitigation (Meinshausen et al., 2020). In combination, these scenarios capture a representative gradient of plausible future climate conditions for assessing species distribution responses under climate change.

Soil variables were characterized using the Harmonized World Soil Database (HWSD; FAO, https://www.fao.org/soils-portal/data-hub/soil-maps-and-databases/harmonized-world-soil-database-v20/en/). Topographic attributes, including elevation, slope, and aspect, were obtained from the Geospatial Data Cloud (http://www.gscloud.cn). Anthropogenic impacts were quantified with the Human Footprint dataset (CIESIN, NASA SEDAC, https://data.nasa.gov/dataset/last-of-the-wild-project-version-3-lwp-3-2009-human-footprint-2018-release), from which eight indicators of human pressure in 2009 were extracted, covering extent of built environments, crop lands, pasture lands, population density, night-time lights, railways, roads, and navigable waterways (Venter et al., 2016a, 2016b). These variables serve as proxies to evaluate both the direct and indirect pressures exerted by anthropogenic factors on species and their surrounding environments.

To ensure spatial consistency, all environmental layers were resampled to a resolution of 2.5 arcmin. Variable selection was conducted by jointly considering Spearman’s rank correlations (|R| < 0.8) and contribution values (Pacifici et al., 2017). Final variable selection was further guided by ecological relevance to *A. guttata*, a desert-adapted medicinal species, with particular emphasis on temperature seasonality, precipitation patterns, and soil conditions known to influence its growth and secondary metabolite accumulation. This procedure reduced multicollinearity and spatial autocorrelation among predictors (Elith et al., 2006; Dormann et al., 2013; Hanberry, 2024).

### 2.3 Species distribution modeling

**Figure 2:**
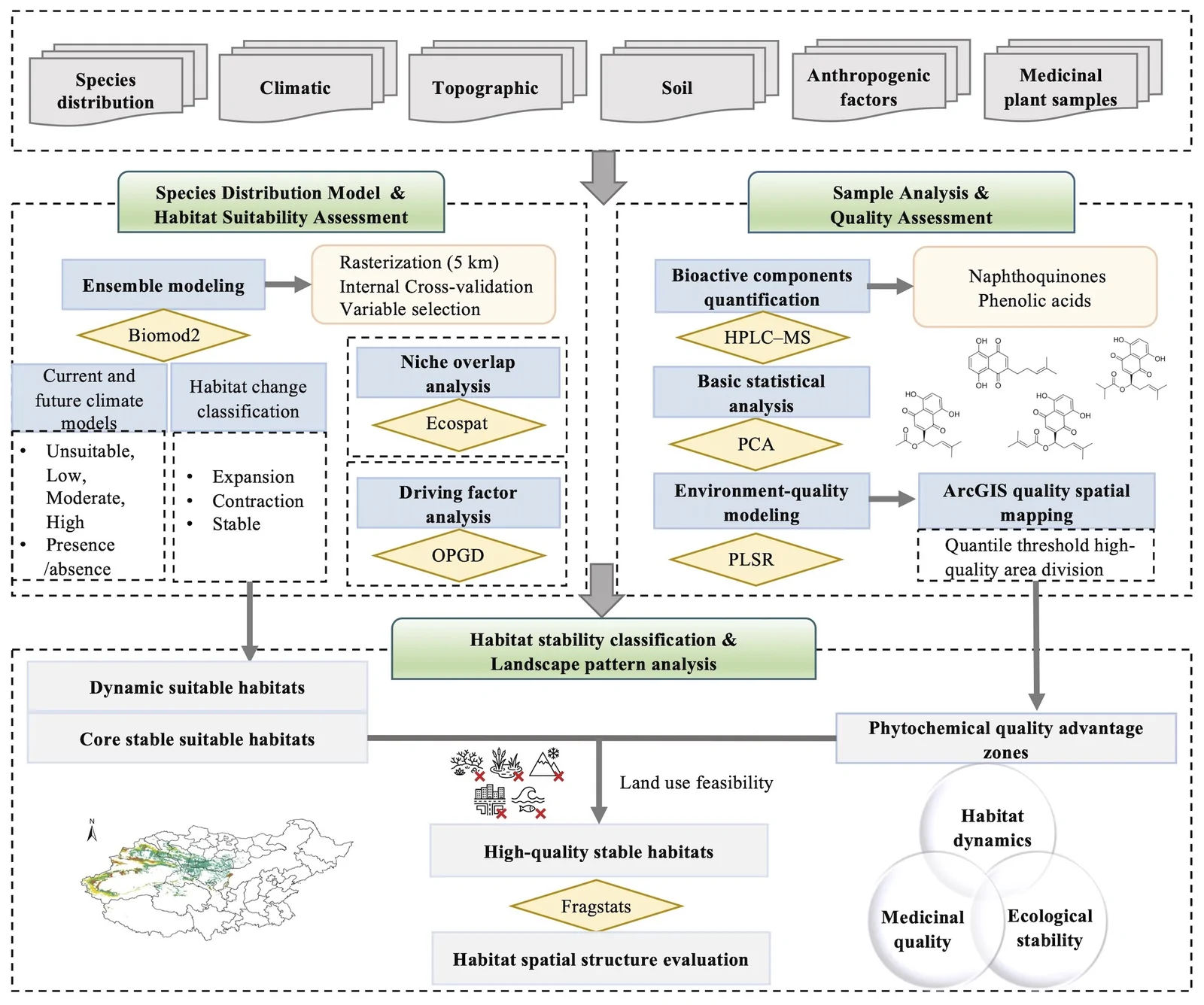
Workflow of model integration for habitat suitability projection of *A. guttata*.

In this study, the biomod2 package (v4.2.5.2) in R was used to integrate eleven algorithms including Artificial Neural Networks (ANN), Classification Tree Analysis (CTA), Flexible Discriminant Analysis (FDA), Generalized Additive Models (GAM), Generalized Boosted Models (GBM), Generalized Linear Models (GLM), Multivariate Adaptive Regression Splines (MARS), Maximum Entropy methods (MAXENT), Random Forests (RF), Surface Range Envelope (SRE), and Extreme Gradient Boosting (XGBOOST) (Phillips et al., 2017). Presence records were combined with pseudo-absence data generated using a random sampling strategy constrained within the accessible area, with 1000 pseudo-absence points produced per run to balance model sensitivity and specificity (Barbet-Massin et al., 2012). To reduce model uncertainty and sampling bias, a repeated split-sample approach was applied, in which 75% of the occurrence records were randomly used for model calibration and 25% for validation, repeated across three independent runs. In addition, a 10-fold cross-validation procedure was implemented within the training dataset to further assess model robustness and stability (Araújo and New, 2007). Model performance was evaluated using the area under the receiver operating characteristic curve (AUC) and the true skill statistic (TSS), and only models with AUC > 0.9 and TSS > 0.7 were retained for subsequent ensemble forecasting (Cao et al., 2024). The final ensemble model was constructed using a weighted mean approach (EMmean), where individual models were weighted according to their evaluation scores (TSS), thereby improving predictive accuracy and reducing algorithm-specific uncertainty (Thuiller et al., 2009) (**Figure 2**).

### 2.4 Current and future habitat suitability patterns

The ensemble model outputs were used to estimate the occurrence probabilities of *A. guttata* for each grid cell, with values ranging from 0 (unsuitable) to 1 (highly suitable). Based on these probabilities, the natural breaks classification method was adopted to categorize habitats into four suitability levels: highly suitable, moderately suitable, low suitability, and unsuitable. Distributional shifts under future climate scenarios were quantified and visualized using the ArcGIS (Brown, 2014).

When evaluating changes in habitat suitability under current and future environmental conditions, occurrence probabilities of *A. guttata* were converted into binary presence-absence maps to facilitate comparative analyses. Changes in habitat extent were then categorized into three responses: habitat contraction, reflecting reductions in suitable habitats; habitat expansion, indicating the emergence of new suitable regions; and habitat stability, where suitability patterns remained largely unchanged. The areas of contraction, expansion, and stable suitable habitats were subsequently calculated to summarize potential dynamics in habitat suitability.

### 2.5 Niche overlap analysis

Ecological niche shifts of *A. guttata* under current and future climate scenarios were analyzed using the PCA-env framework in the R package ecospat (version 4.4.1; Broennimann et al., 2012; Di Cola et al., 2017). To quantitatively test niche conservatism, 10,000 points were selected as its back-ground data. Then environment data, suitability values and binarization results were extracted for each background point. The quantified niche of a species in climate space is represented by some niche indices, including Schoener’s D (*D*), the Hellinger based I (*I*) statistic, niche equivalency, and niche similarity. *D* and *I* statistic were all applied to quantify niche overlap, with values approaching 0 denoting absence of overlap and values near 1 denoting full overlap (Schoener, 1968; Warren et al., 2008). The niche equivalency test evaluates whether the ecological niches of the native and invaded ranges are identical. Occurrence records were randomly reassigned 1000 times, and the observed *D* value was compared at a significance level of 0.05. This test statistically determines whether the observed overlap is significantly higher than random expectations, supporting niche conservatism. The niche similarity test, examines whether differences in environmental space reflect true ecological divergence rather than merely the variation in available environmental conditions between regions. The analysis also used a 0.05 significance threshold to assess the observed overlap against randomly generated expectations (Warren et al., 2010; Aguirre-Gutiérrez et al., 2015; Zuo et al., 2023).

### 2.6 Environmental drivers of habitat suitability

The optimal parameter-based geographical detector (OPGD) framework was applied to quantify the relative contributions and interaction effects of environmental variables on the habitat suitability of *A. guttata* (Wang et al., 2016; Song et al., 2020; Shi et al., 2023). Habitat suitability was used as the dependent variable, and key environmental factors were treated as independent variables. The factor detector was used to assess the explanatory power of each variable for the spatial heterogeneity of habitat suitability, which is quantified by the q-statistic (ranging from 0 to 1), where higher values indicate stronger explanatory power. Continuous variables were discretized using multiple classification schemes, and the scheme yielding the highest *q* value was selected for analysis. The interaction detector was used to compare the explanatory power of single factors with that of paired factors, allowing the identification of interaction types and the strength of their joint effects on habitat suitability. In addition, the risk detector and ecological detector were applied to identify suitable threshold ranges of key variables and to examine differences in habitat suitability across environmental gradients, thereby clarifying the mechanisms through which major drivers shape the spatial pattern of suitable habitats for *A. guttata*.

### 2.7 Quantification of bioactive components and correlation analysis with environmental factors

#### 2.7.1 Quantification of bioactive constituents in *A. guttata*

Root materials of *A. guttata* were dried at 50 °C. For each sample, 0.2 g of powdered root was accurately weighed and extracted with 20 mL of 70% methanol by sonication (40 kHz, 100%) for 30 min at room temperature. After cooling, the extract was brought to the initial weight, filtered, and 2 mL of filtrate was transferred to a centrifuge tube and centrifuged at 13,000 rpm for 10 min. The supernatant was collected, transferred to a new tube, and centrifuged again under the same conditions. The final supernatant was filtered through a 0.22 μm membrane prior to injection.

Major naphthoquinones and phenolic acids, including (-)-shikonin, deoxyshikonin, acetylshikonin, *β,β*′-dimethylacryloylalkannin, isobutyrylshikonin, caffeic acid, rosmarinic acid, and lithospermic acid B, were quantified using authenticated reference standards supplied by Shanghai Yuanye Bio-Technology Co., Ltd. and Shanghai Tauto Biotech Co., Ltd. (purity ≥98%, HPLC).

Analysis was performed on a Shimadzu LC-30AD HPLC system coupled with a high-resolution mass spectrometer operating in information-dependent acquisition (IDA) mode. Electrospray ionization (ESI) was performed in negative mode. Chromatographic separation was achieved on a Caprisil C18-X column (150 mm × 2.1 mm, 1.8 µm) using LC-MS-grade acetonitrile (A) and 0.1% formic acid in water (B) as mobile phases, with a gradient of 20-48% A over 8 min, followed by a further increase to 60% A over 12 min. Method validation included assessments of linearity, precision, repeatability, stability, and recovery, with all calibration curves showing good linearity (R² > 0.999). These results ensured the reliability and reproducibility of quantitative measurements used for subsequent spatial analyses.

#### 2.7.2 Relationships between phytochemical quality indicators and environmental factors

To explore the relationships between phytochemical quality and environmental conditions in *A. guttata*, the quantified contents of active compounds from 25 sampling sites were integrated with environmental variables extracted at the corresponding locations. The coefficient of variation (CV) was calculated for each compound to assess variability among samples, and correlation analyses were performed to examine interrelationships among phytochemical constituents. Principal component analysis (PCA) was applied separately to each group of compounds to reduce dimensionality and to extract composite indicators representing phytochemical quality. Principal components explaining the majority of the total variance were retained, and weighted composite indices were calculated and subsequently used as phytochemical quality indicators in the following analyses.

Partial least squares regression (PLSR) (Wold et al., 2001) was employed to quantify the relationships between environmental factors and phytochemical quality indicators. Prior to modeling, all environmental variables (X matrix) and chemical quality indicators (Y matrix) were standardized using Z-score transformation to eliminate the influence of different units and scales. The PLSR model was constructed using the plsr function in the pls package in R. The optimal number of latent components was determined using five-fold cross-validation, with the minimum root mean square error of prediction (RMSEP) as the selection criterion, after excluding the intercept-only model. Model performance was evaluated using the coefficient of determination for the training set (R²), cross-validated R² (Q²), and RMSEP. Variable importance in projection (VIP) values were calculated to assess the relative contribution of each environmental variable to the PLSR model.

Based on the final PLSR model, a continuous quality suitability map was generated by applying the regression equation to the corresponding environmental raster layers using the raster calculator in ArcGIS, thereby representing the spatial distribution of predicted phytochemical quality. To delineate phytochemical quality advantage zones, a quantile-based threshold was applied to the quality suitability map, with grid cells in the top 25% classified as high-quality areas. These areas were then overlaid with ENM-derived climatically suitable habitats to identify regions with both phytochemical quality advantages and climatic suitability.

### 2.8 Environmental stability and land use characteristics of the high-quality stable habitats

To further clarify the spatiotemporal stability of suitable habitats for *A. guttata* under current and future climate scenarios, and to provide a basis for planting and conservation planning, suitability maps derived from species distribution modeling under different time periods and emission scenarios were superimposed. Areas consistently predicted as suitable across all scenarios were defined as core stable suitable habitats, whereas regions exhibiting gains or losses of suitability among scenarios were classified as dynamic suitable habitats. Differences in environmental conditions between these two zones were further examined to explore the environmental factors underlying their spatial differentiation. This classification follows a stability-based ecological framework, in which persistent suitability across multiple scenarios is used to represent long-term habitat reliability.

To identify areas of high-quality stable habitats, the phytochemical quality advantage zones were overlaid with the core stable suitable habitats. This step represents a multi-criteria integration process that combines ecological suitability with phytochemical quality to identify priority habitats. Subsequently, these high-quality and stable suitable habitats were intersected with a global land-use dataset (https://doi.org/10.5194/essd-17-4039-2025) to assess land availability and practical feasibility. Land cover types classified as water, permanent ice and snow, and impervious surfaces were excluded, as these land-use types were assumed unsuitable for the natural regeneration or survival of *A. guttata*. By further incorporating land-use constraints, the final delineation outcome a hierarchical habitat evaluation framework that integrates environmental suitability, medicinal quality, and realistic land-use feasibility. The remaining areas were therefore considered potentially available habitats with both high-quality stable habitats.

### 2.9 Landscape pattern analysis of different habitat types

To further quantify the spatial structure of different habitat types, landscape pattern analysis was conducted using Fragstats 4.2. Three habitat types were evaluated: core stable suitable habitats, phytochemical quality advantage zones, and high-quality stable habitats. All rasters were resampled to a consistent resolution and converted to binary maps prior to analysis. Three commonly used landscape metrics were selected to characterize fragmentation and spatial configuration, including patch density (PD), largest patch index (LPI), and aggregation index (AI), which respectively reflect landscape fragmentation, dominance, and aggregation patterns (Riitters et al., 1995; Turner, 2005; McGarigal et al., 2012). Comparative analysis was conducted to identify differences in fragmentation and spatial configuration among the three habitat types, thereby providing a basis for targeted conservation and sustainable utilization.

## 3 Results

### 3.1 Model accuracy evaluation

In the study of *A. guttata*, all eleven modeling algorithms were successfully implemented, yielding 330 prediction results. The models were evaluated using ROC and TSS as performance criteria. Random Forest provided the most accurate predictions for *A. guttata*, while GBM, XGBoost, MARS, and MaxEnt also showed strong performance (**Table 1**). In contrast, the artificial neural network performed the worst and failed to reach the required accuracy threshold. After filtering, models with ROC values greater than 0.9 and TSS values greater than 0.7 were selected for ensemble construction, while retaining as many model features as possible. A total of 264 results were used to construct the ensemble model of *A. guttata*. The ensemble model for *A. guttata* reached a TSS of 0.797 and an ROC of 0.962, indicating robust predictive reliability for species. These models provide a reliable basis for assessing habitat suitability under future climate scenarios.

**Table 1:** TSS and AUC values across 11 models.

Models	ROC_mean	ROC_SD	TSS_mean	TSS_SD
RF	0.9999	0.0003	0.9912	0.0039
GBM	0.9745	0.0023	0.8647	0.0092
XGBOOST	0.9465	0.0057	0.7953	0.0219
MARS	0.9472	0.0032	0.7723	0.0150
MAXENT	0.9464	0.0040	0.7595	0.0143
GLM	0.9367	0.0053	0.7515	0.0155
FDA	0.9347	0.0052	0.7396	0.0135
ANN	0.9247	0.0166	0.7565	0.0361
GAM	0.9132	0.0051	0.6934	0.0159
CTA	0.9095	0.0271	0.7807	0.0527
SRE	0.7707	0.0118	0.5416	0.0237

### 3.2 Suitable habitats for *A. guttata* currently

Under the current climate, the ensemble model indicated that the potential habitats of *A. guttata* were mainly distributed in northwestern China, including the Altai Mountains, Tianshan Mountains, and Kunlun Mountains, also occurred widely in southern Mongolia, with concentrated distribution in the southwestern Mongolian Plateau.

**Figure 3:**
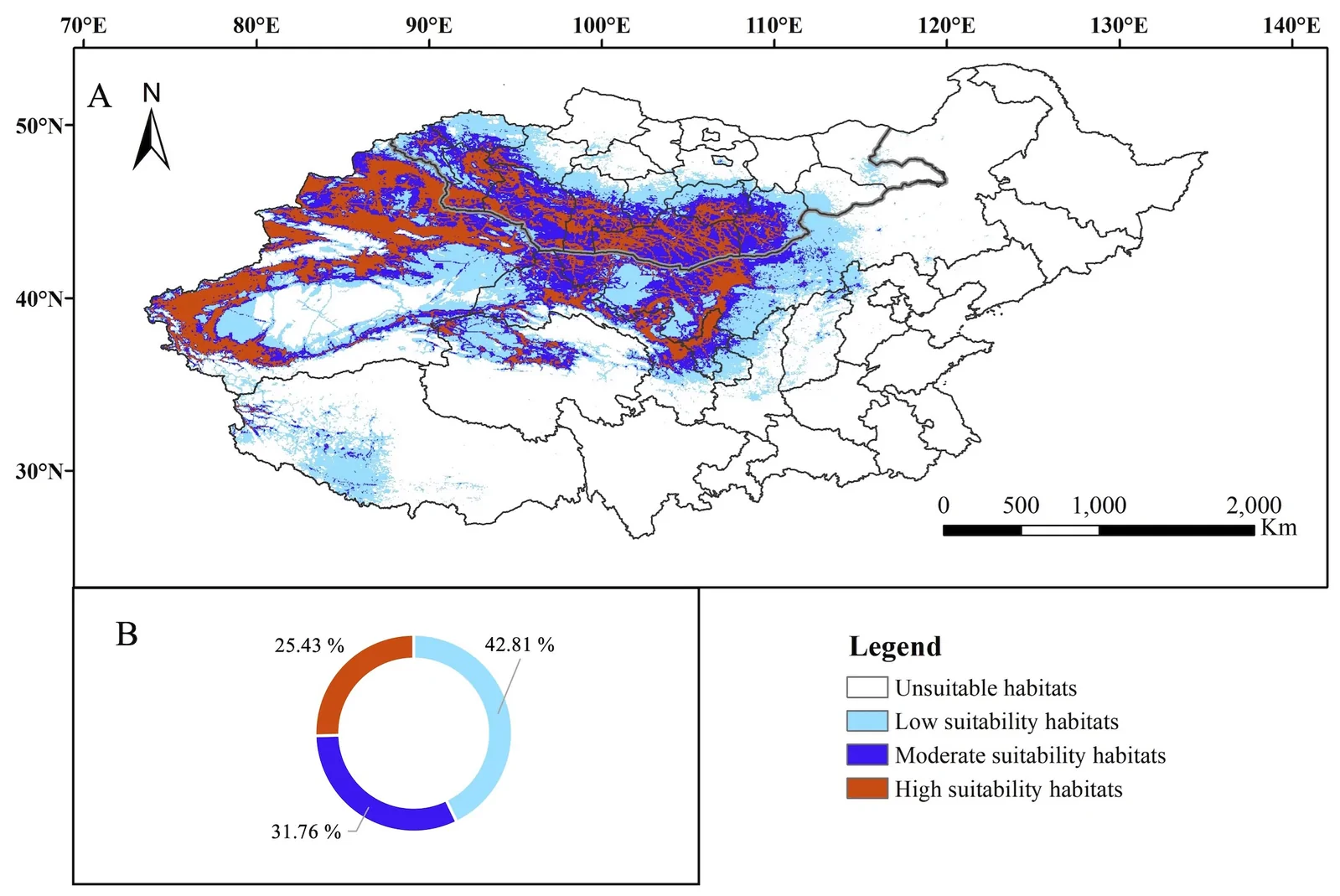
Suitable habitats of *A. guttata* relative to the current climate scenarios. **(A)** Spatial distribution of suitable habitats; **(B)** Proportional composition of suitability classes.

The suitable habitats of *A. guttata* were about 359.48 ×10^4^ km², with 74.77% distributed in China and 25.23% in Mongolia. In China, high suitable habitats occupy about 68.18 ×10^4^ km², with 75.16 ×10^4^ km² classified as moderate suitability, 125.44 ×10^4^ km² as low suitability. In Mongolia, high suitable habitats cover approximately 23.20 ×10^4^ km², while moderate- and low-suitability areas account for 37.51 ×10^4^ km², 29.99 ×10^4^ km², respectively. High-suitability habitats were localized in Xinjiang, Inner Mongolia, and Gansu in China, as well as in western and southern Mongolia, particularly in Bayan-Ölgii, Khovd, Govi-Altai, Bayankhongor, and Dornogovi (**Figure 3**).

### 3.3 Suitable habitats range in the future

**Figure 4:**
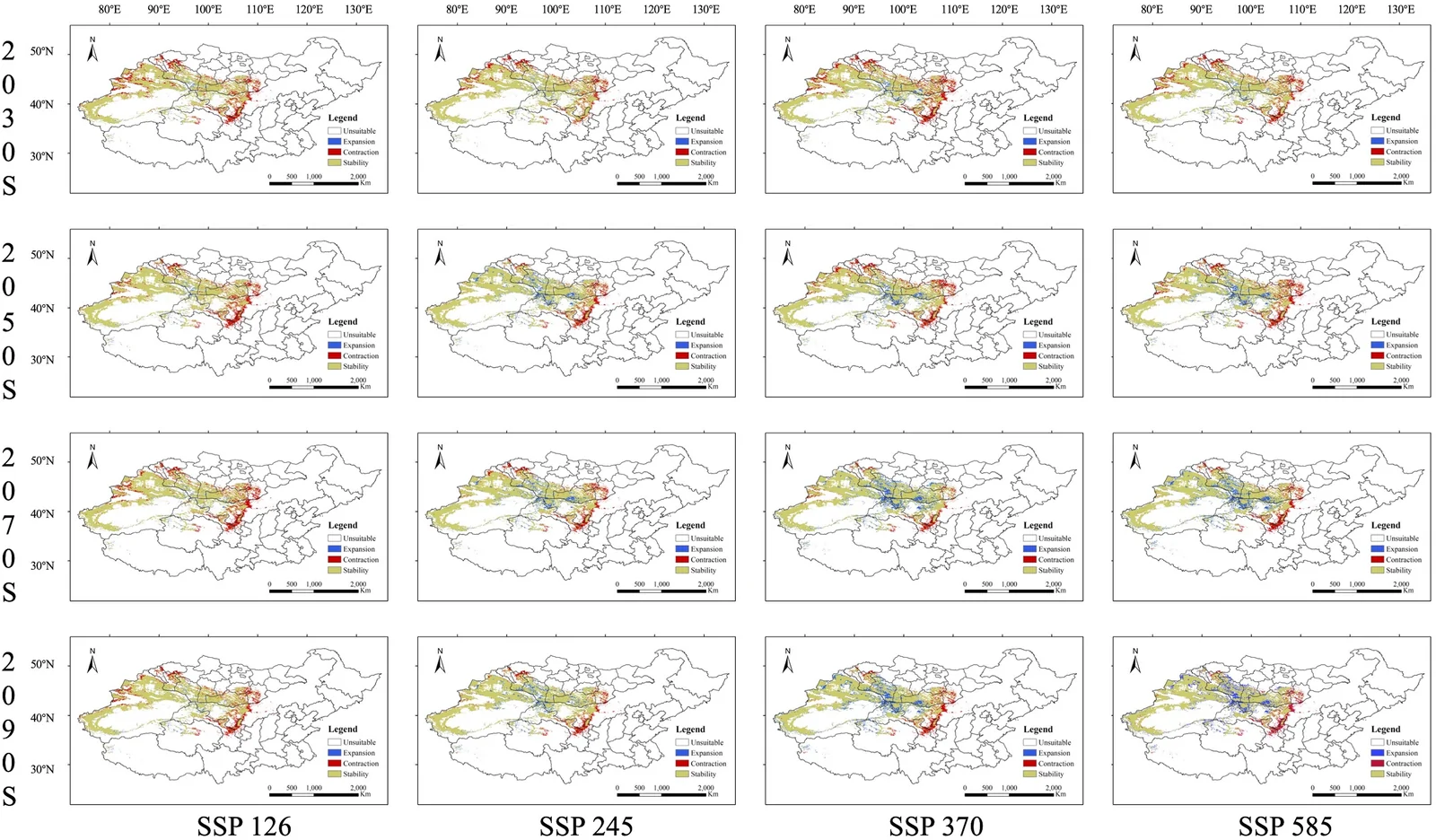
Changes in suitable habitats of *A. guttata* relative to the future climate change scenarios.

**Figure 5:**
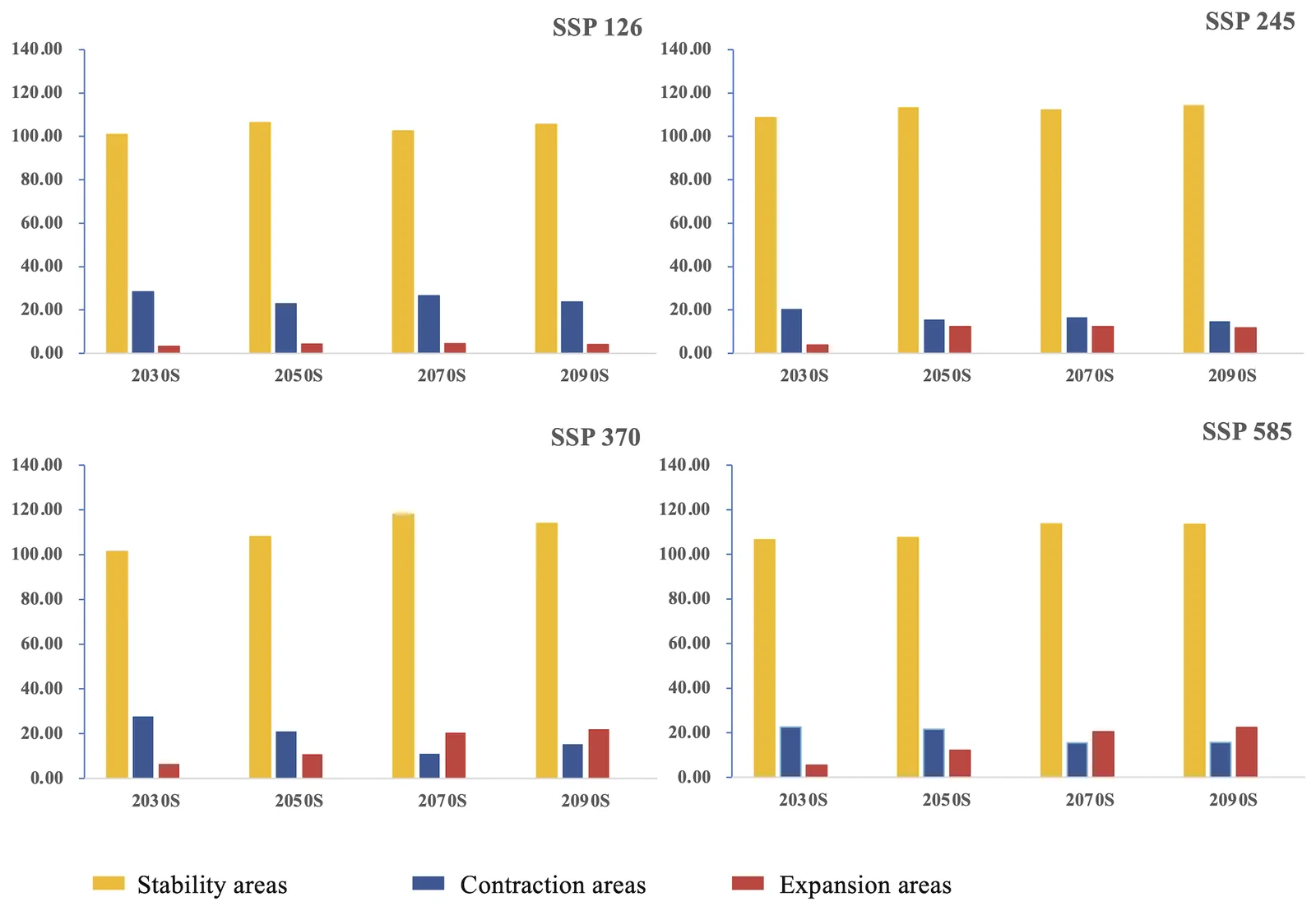
Unchanged, contracted, and expanded suitable habitats of *A. guttata* under various future climate scenarios.

From the present climate to 2100, the potential distribution of *A. guttata* is projected to shift under different climate scenarios (**Figures 4**, **5**). Under the SSP 126 scenario, the suitable habitats remain relatively stable, with only slight fluctuations in contraction and expansion. Across all scenarios, the extent of range contraction consistently exceeds that of expansion.

By contrast, the SSP 245 scenario shows a progressive expansion of suitable habitats. Contraction areas decline to 11.66×10^4^ km² by the 2090s. Habitat expansion reaches its peak in the mid-century (2050s-2070s), with more than 12.31 ×10^4^ km² of newly suitable habitats.

Under the SSP 370 scenario, the most pronounced range shifts are observed. Stable habitats increased substantially, with habitat contraction reduced to 11.06 × 10^4^ km² and newly suitable habitats expanding to 22.0 × 10^4^ km² by the end of the century. This scenario indicates the largest net habitat gain, particularly in the southwestern part of the Mongolian Plateau.

In comparison, under the SSP 585 scenario, contraction and expansion dynamics become more variable. Expansion increases steadily from 5.47 ×10^4^ km² in the 2030s to more than 22.47 ×10^4^ km² in the 2090s, while contraction areas remain around 15.73-22.63×10^4^ km² (**Table 2**).

**Table 2:** Statistical table of changes in the suitability zones of *A. guttata* under future environmental conditions.

Climate scenario	Years	Stability (×10^4^ km²)	Contraction (×10^4^ km²)	Expansion (×10^4^ km²)
SSP 126	2030s	100.87	28.29	3.25
	2050s	106.29	22.88	4.20
	2070s	102.60	26.57	4.44
	2090s	105.54	23.63	4.06
SSP 245	2030s	108.71	20.46	3.88
	2050s	113.51	15.65	12.25
	2070s	112.53	16.64	12.31
	2090s	114.44	14.73	11.66
SSP 370	2030s	101.35	27.81	6.46
	2050s	108.14	21.02	10.81
	2070s	118.10	11.06	20.42
	2090s	113.86	15.31	22.00
SSP 585	2030s	106.53	22.63	5.47
	2050s	107.55	21.62	12.06
	2070s	113.59	15.57	20.41
	2090s	113.43	15.73	22.47

Overall, the SSP 370 scenario suggests the most favorable future trajectory for *A. guttata*, with the largest net expansion and stabilization of suitable habitats, while the SSP 585 pathway indicates a risk of habitat degradation despite some local expansions. Under both scenarios, the expansion of suitable habitats during the 2070s and 2090s is more pronounced than the contraction. The areas of contraction are mainly located along the southwestern margin of the current suitable range, whereas the expansion zones are primarily concentrated in the central part of the original distribution area, particularly in the Altai, Qilian, Helan, and Tianshan Mountains along the China-Mongolia border. Despite these spatial shifts in suitable habitats, the distribution centroid of *A. guttata* consistently remained in Yizhou District, Xinjiang, northwestern China, showing no evident displacement across different climate scenarios and time periods. These contrasting responses reveal substantial differences in habitat expansion and contraction patterns among future climate scenarios.

### 3.4 Climatic niche overlap, equivalency, and similarity

**Figure 6:**
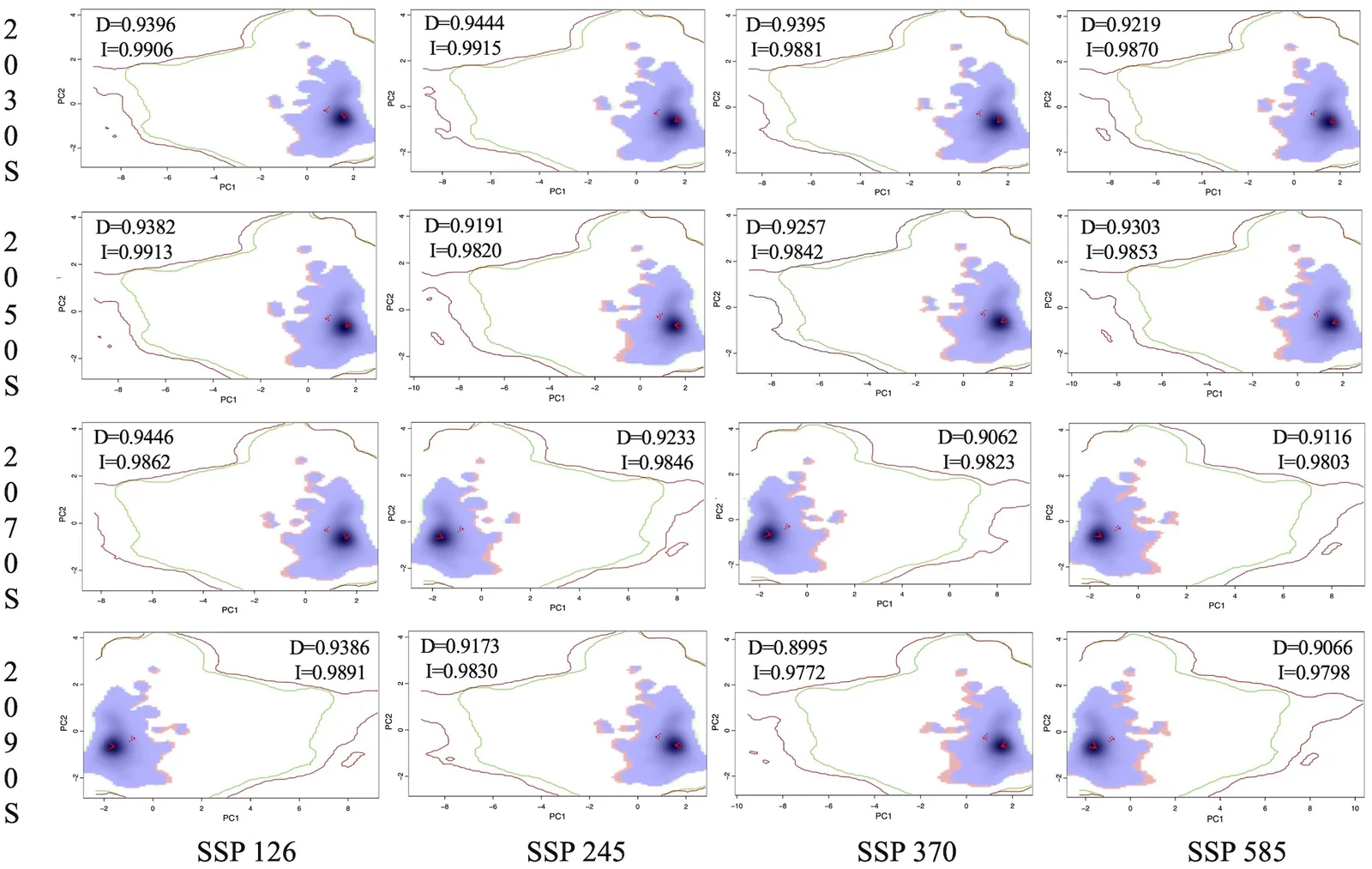
Niche overlap of *A. guttata* under current and further climate scenarios.

Ecological niche comparisons using the ecospat framework showed a high overlap between the current and future potential distributions of *A. guttata*. Niche overlap indices remained consistently high, with *D* values ranging from 0.90 to 0.94 and *I* values from 0.97 to 0.99. These results indicate strong niche conservatism in A. guttata. Most future suitable habitats remained within the current climatic niche, suggesting that projected range shifts mainly represent geographic redistribution rather than adaptation to novel environmental conditions. Although niche equivalency and similarity tests were significant (p < 0.05), only minor spatiotemporal differences were detected among scenarios. As shown in **Figure 6**, areas of niche overlap dominate, while limited niche expansion and unfilling occur along specific climatic gradients. The direction of niche shifts varied among scenarios, with a general tendency toward the southwest, and occasional southeastward shifts under selected mid- and late-century scenarios. These patterns imply that climate gradients may facilitate range expansion or redistribution of *A. guttata*, rather than forcing a contraction of its climatic niche.

### 3.5 Environmental drivers of suitability

In this study, eight environmental variables (BIO2, BIO4, BIO9, BIO16, BIO18, D2_BSAT, ELE, and HF) included in the final ensemble model were used to analyze the driving mechanisms of habitat suitability. The OPGD analysis enhanced the standard geographical detector framework by incorporating multiple discretization methods and interval classifications.

The factor detector results showed that q values of environmental variables ranged from 0.0445 to 0.5425, demonstrating clear differences in their contributions to the spatial heterogeneity of habitat suitability. Among all variables, BIO18 had the highest explanatory power, followed by BIO16 and D2_BSAT, indicating that precipitation and soil properties were the primary factors associated with the distribution of *A. guttata.* Both BIO18 and BIO16 mainly occurred within the 0–500 mm range, suggesting that water availability during the growing season strongly constrains species distribution. D2_BSAT represents soil base saturation, defined as the proportion of exchangeable cations (Na^+^, Ca²^+^, Mg²^+^, and K^+^) relative to total cation exchange capacity (Batjes, 2016). Most values were distributed within the 80–100 range, accounting for more than 80% of the total frequency, which reflects the preference of *A. guttata* for soils with relatively high base saturation.

**Figure 7:**
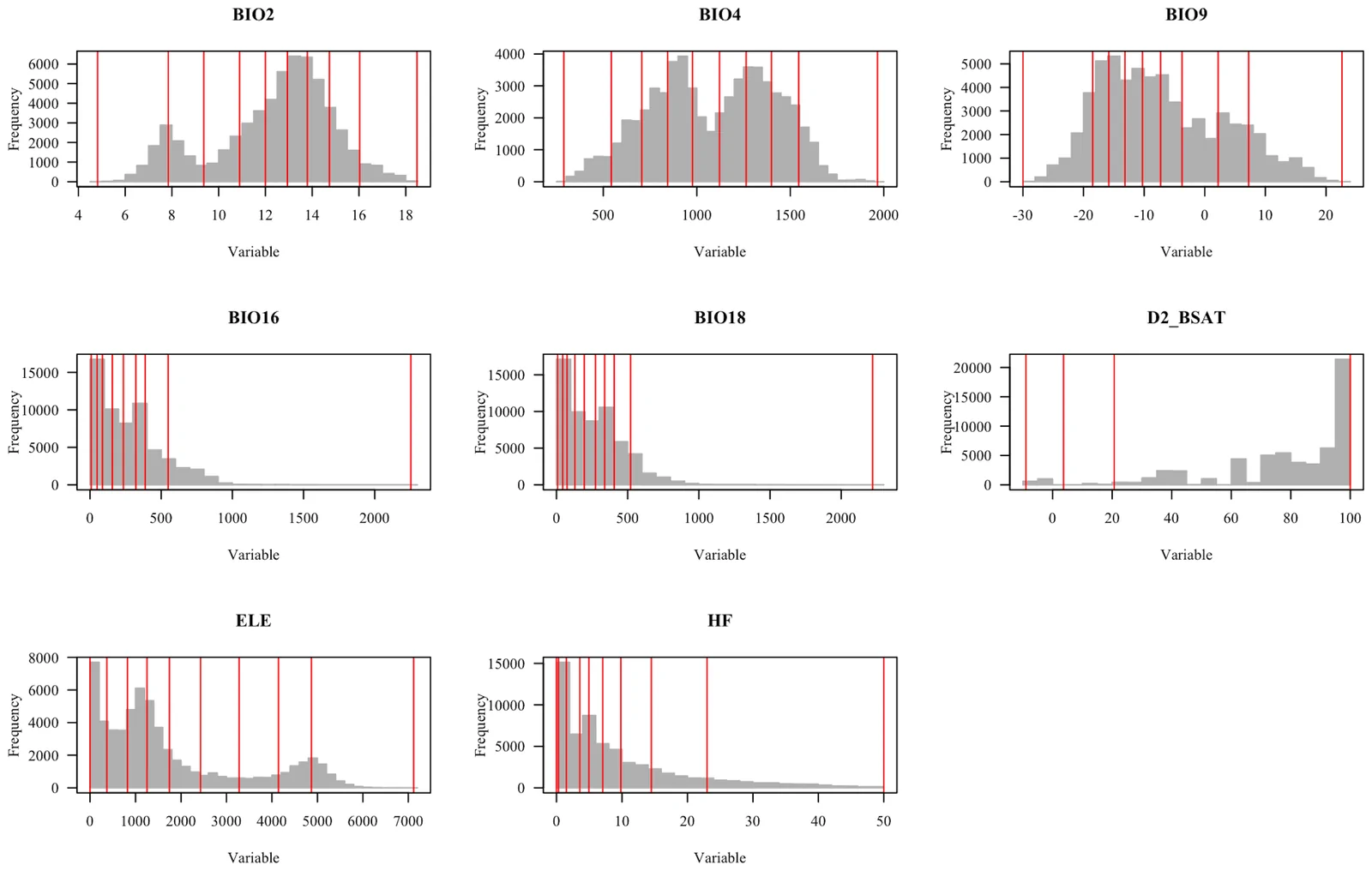
Variable frequency distribution and identification of anomalous intervals based on OPGD.

Temperature-related variables (BIO2, BIO4, and BIO9) showed moderate effects on habitat suitability. BIO2 mainly ranged from 10-16 °C, BIO4 (standard deviation ×100) from 800-1500, and BIO9 from -20 to 5 °C, reflecting the species’ tolerance to relatively cold conditions and broad temperature variability. Suitable habitats were distributed across a wide elevational gradient, although most occurred below 2000 m. In addition, low HF values indicate that *A. guttata* is mainly distributed in areas with relatively weak human disturbance. Overall, precipitation-related variables played the dominant role in determining broad habitat distribution, whereas soil and topographic conditions contributed more to local-scale habitat variation (**Figure 7**).

**Figure 8:**
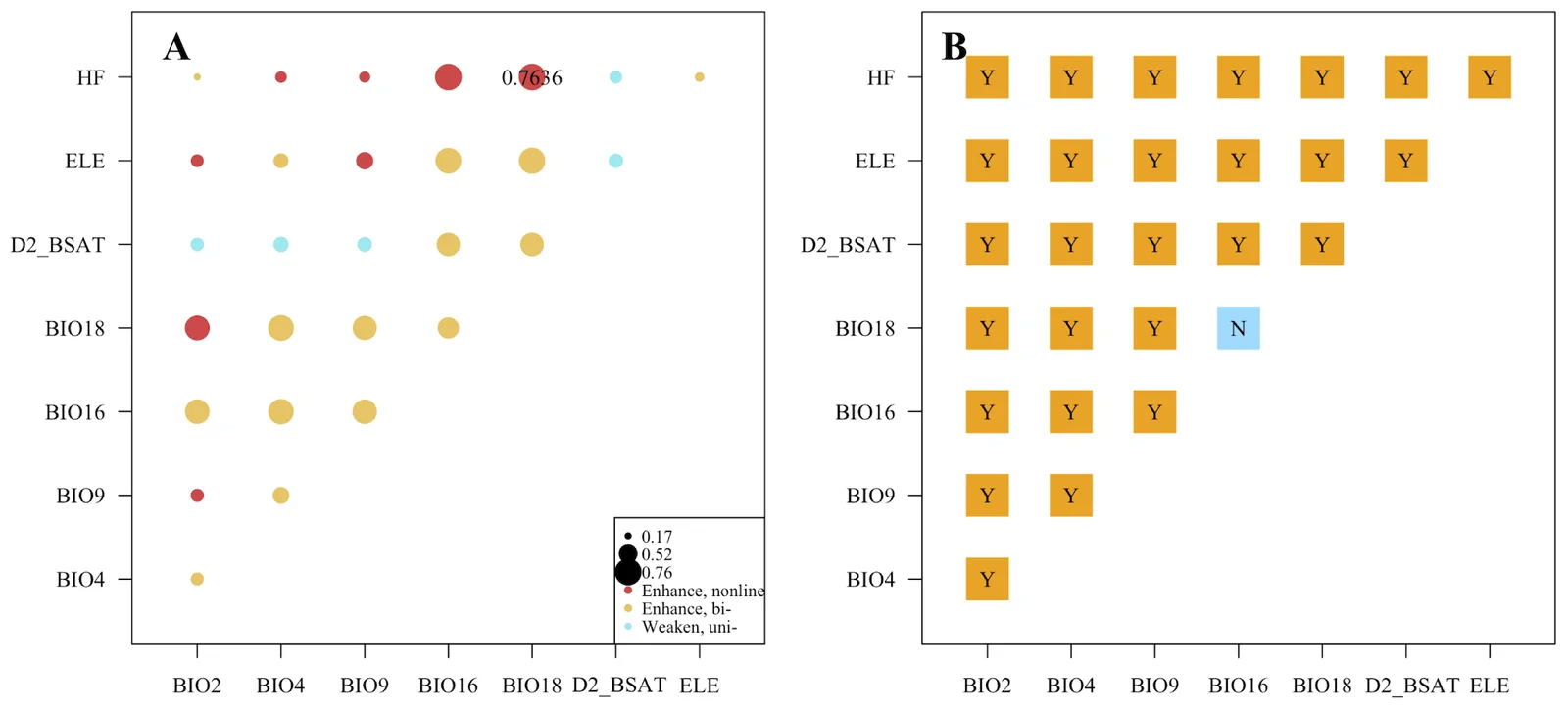
Visualization of interaction and ecological detector results derived from the OPGD framework. **(A)** Interaction strength of environmental variable; **(B)** Factor detection significance of environmental variables.

Interaction detector results revealed significant nonlinear-enhance interactions between BIO18 and HF, and between BIO16 and HF. This indicates that in regions with abundant or variable precipitation, human activities can amplify their effects on species distribution and habitat stability. BIO18 and BIO16 also showed significant bi-enhance effects with ELE, BIO2, BIO4, and BIO9, highlighting strong bidirectional interactions among precipitation, temperature, and elevation that shape habitat suitability. No significant interaction was detected between BIO18 and BIO16, suggesting that these precipitation variables operate largely independently. In contrast, D2_BSAT showed weakening effects with BIO2, BIO4, BIO9, HF, and ELE, indicating that soil base saturation has weaker combined effects and acts more independently in regulating species suitability (**Figure 8**).

In general, the interaction between precipitation and human disturbance was the primary factor driving spatial heterogeneity in habitat suitability. Bidirectional interactions between temperature and elevation were secondary, while soil factors played a supporting role at the local scale. These results reveal the complex interplay of climatic, topographic, soil, and anthropogenic factors and provide insights into the ecological mechanisms influencing *A. guttata* under future climate change.

### 3.6 Multivariate statistical analysis of phytochemical constituents

**Figure 9:**
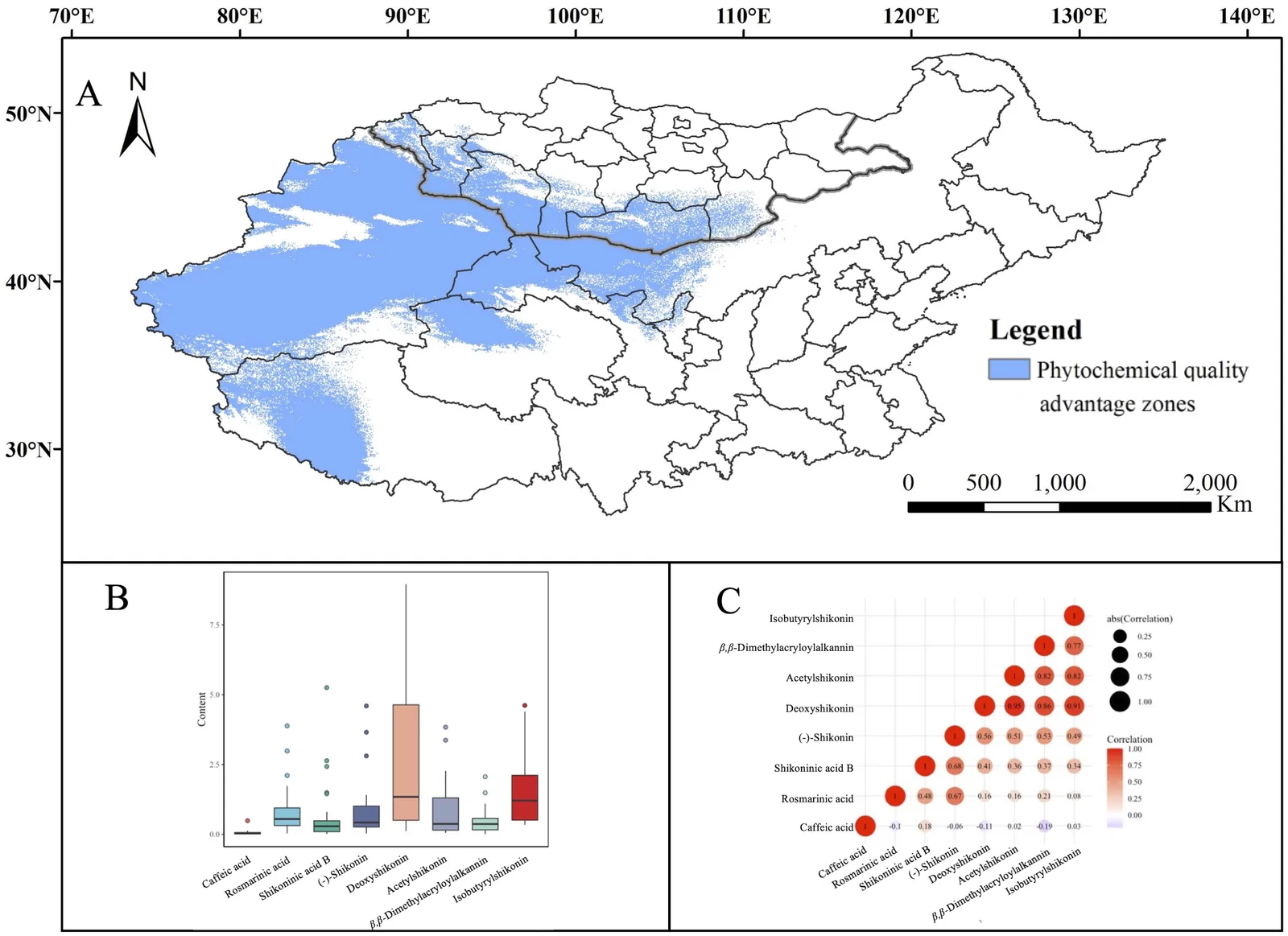
Phytochemical quality patterns of *A. guttata.* **(A)** Phytochemical quality advantage zones; **(B)** Boxplots of phytochemical consituents contents; **(C)** Spearman correlation analysis among phytochemical consituents.

The concentrations of eight representative bioactive compounds, including (-)-shikonin, deoxyshikonin, acetylshikonin, *β,β*′-dimethylacryloylalkannin, isobutyrylshikonin, caffeic acid, rosmarinic acid, and lithospermic acid B, were quantified in 25 batches of *A. guttata* samples (**Supplementary Table 3**). The coefficients of variation (CVs) ranged from 77.99% for isobutyrylshikonin to 164.52% for lithospermic acid B, reflecting pronounced spatial variation in phytochemical composition. Among these compounds, deoxyshikonin and acetylshikonin generally showed relatively high contents and large differences among sampling batches (**Figure 9B**).

Correlation analysis further revealed contrasting relationships among the detected compounds. Deoxyshikonin, acetylshikonin, *β,β*′-dimethylacryloylalkannin, and isobutyrylshikonin exhibited strong positive correlations, with most correlation coefficients above 0.5. Samples with higher concentrations of one of these compounds generally also contained elevated levels of the others. Deoxyshikonin was particularly closely associated with acetylshikonin and isobutyrylshikonin, consistent with similarities in biosynthetic processes. In contrast, caffeic acid showed weak or negative correlations with most other compounds, and its accumulation pattern differed from that of the major naphthoquinone derivatives. Rosmarinic acid showed moderate positive correlations with lithospermic acid B and (-)-shikonin, whereas the relationships among phenolic acid-related compounds were comparatively less consistent (**Figure 9C**). These findings indicate naphthoquinone derivatives form a cohesive group with coordinated accumulation, while phenolic acids exhibit more variable and independent accumulation profiles.

The PLSR model was applied to explore the broad-scale relationships between phytochemical quality and environmental gradients in *A. guttata*. The PLSR model exhibited moderate explanatory power (R² = 0.4292) and acceptable predictive ability (Q² = 0.3594), indicating a reasonable relationship between predictors and response variables. The RMSEP value (0.4241) further suggested an acceptable prediction error. Although the model performance was not strong, it was sufficient for capturing general trends and supporting exploratory analysis. Environmental variables explained only part of the observed variation in phytochemical composition, implying that intrinsic metabolic regulation, genetic differentiation, and microhabitat heterogeneity may also contribute substantially to phytochemical accumulation. VIP analysis identified precipitation during the warm season (BIO18) and soil clay content (D2_CLAY) as the most influential environmental factors associated with phytochemical quality variation, whereas the remaining variables showed comparatively lower contributions. Based on the final PLSR model, the following regression equation was derived to describe the relative associations between environmental variables and phytochemical quality:

(1) Prediction = 1.7416 − 0.0013*ASP − 0.0064*BIO15 − 0.0067*BIO18 + 0.0284*BIO9 − 0.0014*D2_BSAT + 0.0167*D2_CLAY − 0.0234*D2_CN_RATIO+ 0.0066*D2_COARSE− 0.0017*D2_ESP

The equation (Equation 1) was subsequently applied to the environmental raster layers to map the relative spatial variation in predicted phytochemical quality across the study region. In this study, phytochemical quality advantage zones represent areas with relatively high integrated phytochemical quality, as predicted by the PLSR-based quality suitability model. Compared with the broader distribution of climatically suitable habitats, phytochemical quality advantage exhibited a more spatially clustered pattern and covered a larger overall area, with several concentrated hotspots identified in environmentally favorable regions (**Figure 9A**). The phytochemical quality advantage zones were then overlaid with the core stable suitable habitats derived from species distribution modeling. Areas shared by both layers were regarded as high-quality stable habitats, reflecting regions that simultaneously possess relatively favorable phytochemical characteristics and long-term environmental stability.

### 3.7 Environmental stability and land-use constraints of the suitable habitats for *A. guttata*

**Figure 10:**
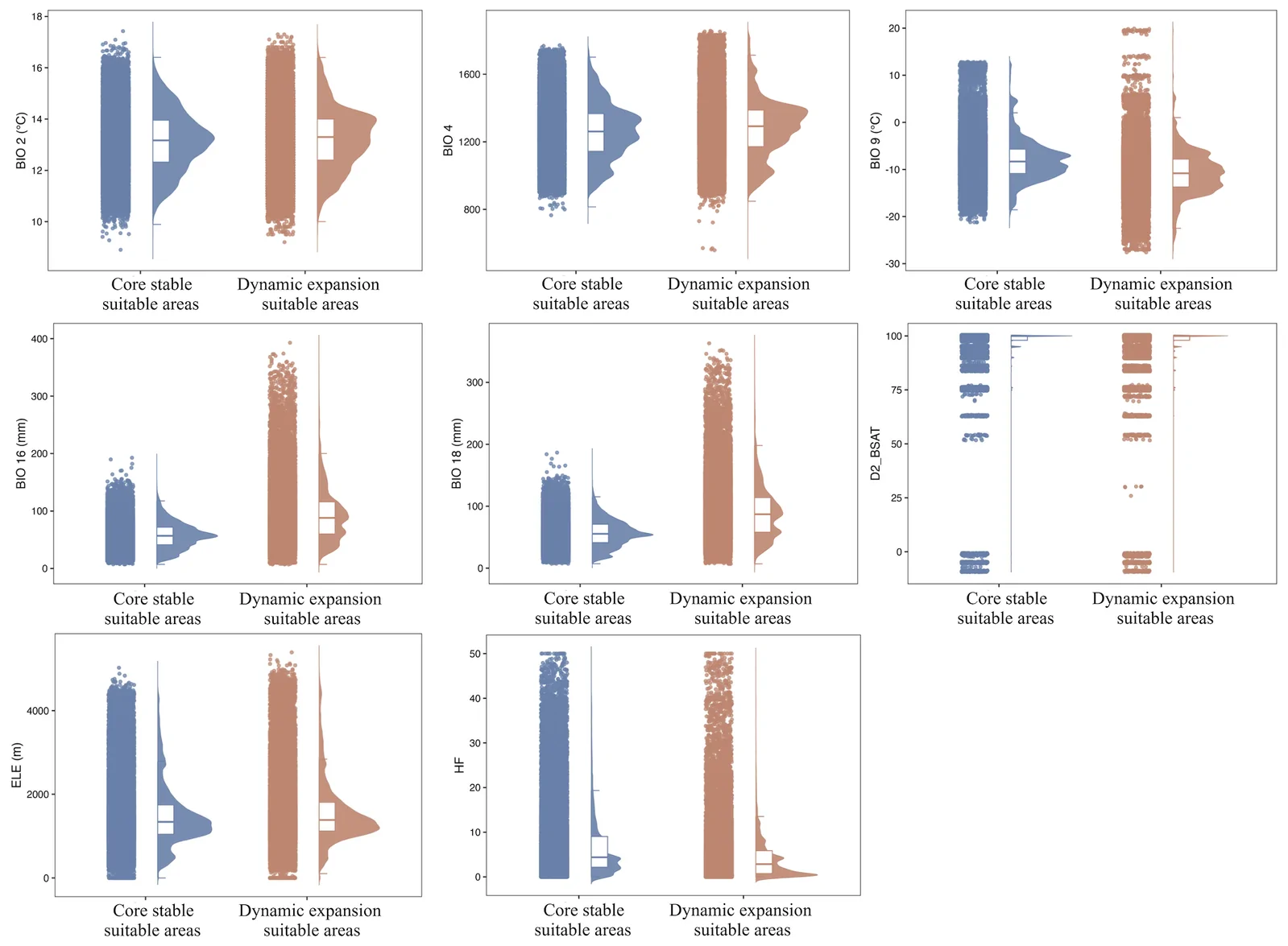
Environmental stability between the core stable suitable habitats and the dynamic suitable habitats.

Environmental variables were compared between core stable and dynamic suitable habitats under different climate scenarios (**Figure 10**). Among the examined variables, BIO16, BIO18, and BIO9 showed the clearest differences between the two habitat types. Core stable suitable habitats generally occupied narrower climatic ranges with lower fluctuations in precipitation- and temperature-related variables. In contrast, dynamic suitable habitats covered broader environmental gradients, including wider precipitation ranges, stronger variation in warm-season temperature, and a larger elevational span. Newly emerging suitable areas under future climate scenarios therefore tended to occur under more variable environmental conditions than the climatically stable regions.

Building on these findings, land-use characteristics of the core stable suitable habitats were further analyzed (**Supplementary Table 2**). The results showed that these areas were predominantly composed of bare land (62.85%), grassland (20.58%), and cropland (11.15%), with bare land as the dominant land-use type. This pattern reflects the species’ preference for open, sparsely vegetated, and desertified environments, consistent with its drought tolerance and adaptation to sandy or gravelly soils. The relatively high proportion of grassland further indicates adaptability to arid and semi-arid steppe ecosystems.

Notably, the dominant land-use types within core stable suitable habitats are generally associated with low-intensity human disturbance, which is consistent with the weak contribution of human activity observed in the driving factor analysis. In this context, land use functions more as an anthropogenic filter than as a primary driver of species distribution. These dominant land-use types are also consistent with the ecological preferences of *A. guttata* for open habitats with abundant solar radiation and well-drained soils. For cultivation and conservation planning, land-use types such as tundra, wetland, permanent ice and snow, impervious surfaces, and water bodies should be excluded due to ecological and practical constraints. However, these unsuitable land-use categories account for only a small proportion of the area. The persistence of *A. guttata* is closely associated with low temporal variability in key climatic variables and a low-disturbance land-use background. The core stable suitable habitats therefore exhibit both climatic stability and land-use stability, providing favorable conditions for the long-term persistence of *A. guttata* populations.

### 3.8 Landscape pattern characteristics of different habitat types

Landscape metrics showed clear differences in spatial configuration among the three habitat types (**Table 3**). Core stable suitable habitats, representing areas consistently predicted as suitable across multiple periods, had very low patch density (PD = 0.0001), a relatively small largest patch index (LPI = 6.34), and moderate aggregation (AI = 79.13). These habitats were distributed as scattered patches without a clearly dominant core area. By contrast, phytochemical quality advantage zones also showed low patch density (PD = 0.0001), but their aggregation index increased markedly (AI = 90.55), accompanied by a higher LPI value (23.31). Compared with core stable suitable habitats, these zones were more clustered and continuous distribution pattern, with several large connected patches distributed across the study region.

**Table 3:** Landscape pattern metrics of different habitat types for *A. guttata*.

Suitable habitats	PD	LPI	AI
Core stable suitable habitats	0.0001	6.3376	79.1315
Phytochemical quality advantage zones	0.0001	23.3138	90.5470
High-quality stable habitats	0.0012	55.4272	79.4190

After overlaying the two layers and excluding unsuitable land-use types, high-quality stable habitats displayed a marked increase in patch density (PD = 0.0012), accompanied by a sharply elevated LPI = 55.43) and moderate aggregation (AI = 79.42). The increase in PD indicates a greater number of habitat patches in the landscape. However, the high LPI value suggests that more than half of the habitat area remained concentrated in a single dominant patch. Compared with phytochemical quality advantage zones, aggregation decreased, but remained comparable to that of the core stable suitable habitats. Multi-criteria screening and land-use constraints substantially reshaped habitat configuration, resulting in a spatial pattern characterized by a dominant core patch accompanied by multiple smaller habitat fragments.

**Figure 11:**
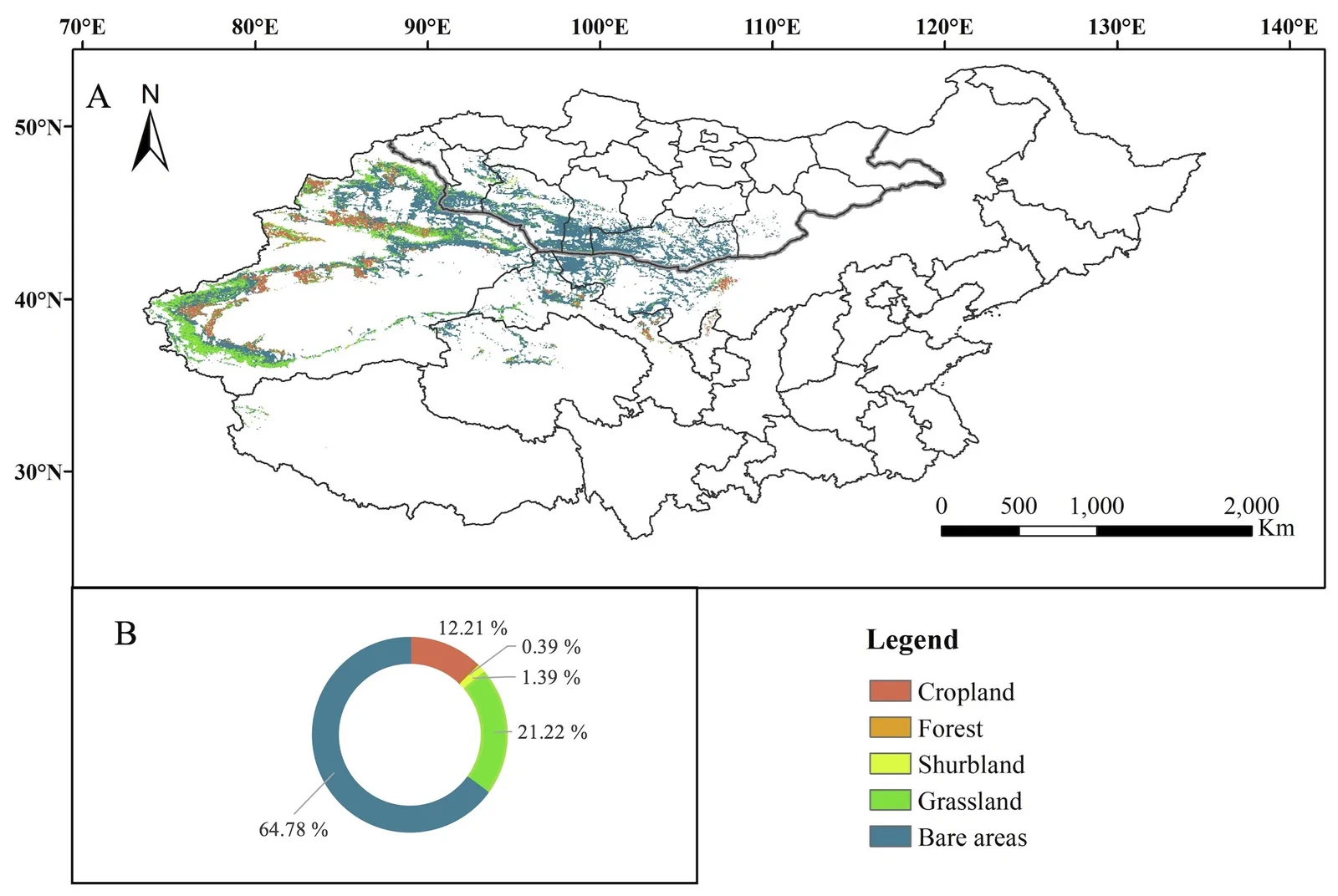
Land use patterns within the high-quality stable habitats. **(A)** Spatial distribution of land-use types; **(B)** Proportional composition of land-use categories.

From a spatial perspective, core stable suitable habitats were distributed as relatively dispersed patches, whereas phytochemical quality advantage zones exhibited greater spatial aggregation and continuity. High-quality stable habitats represented the overlap between climatically suitable areas and regions favorable for phytochemical accumulation. The dynamically expanding suitable habitats projected for future climate scenarios failed to fall within the scope of high-quality stable habitats. This mismatch suggests newly formed climate-suitable zones rarely overlap with areas that yield high levels of active ingredients. Consequently, high-quality stable habitats occupied a more restricted spatial range and were mainly concentrated in the Altai, Qilian, Helan, and Tianshan Mountains (**Figure 11**).

## 4 Discussion

### 4.1 Climate adaptation and distribution shifts

Global warming and changes in precipitation patterns have significantly reshaped the potential distribution of plants (IPCC, 2021). Our projections suggest that *A. guttata* does not respond uniformly to future climate change, but instead exhibits scenario-dependent distribution dynamics across emission pathways. Compared to other threatened medicinal plants (Li et al., 2013), the outlook for *A. guttata* appears relatively stable. Under the low-emission scenario SSP126 during 2020-2040, suitable habitats are projected to contract slightly. Under the more extreme scenarios of SSP370 and SSP585 in the late 21st century, the species exhibits a more dispersed pattern of spatial expansion. The Altai, Qilian, Helan, and Tianshan Mountains may function as climatic refugia, where topographic heterogeneity buffers thermal and hydrological stress and supports long-term population persistence (Dobrowski, 2011; Chen et al., 2011; Keppel et al., 2012). In arid and semi-arid regions of northwestern China and Mongolia, projected increases in warm-season precipitation may partially offset thermal stress, thereby creating new opportunities for species persistence and expansion (Yan et al., 2022). The core stable suitable habitats show low climate displacement and stable environments, supporting their prioritization for *in situ* conservation, germplasm preservation, and future cultivation or restoration.

Despite limited sampling caused by the threatened status of the species, PLSR captured relative spatial gradients in phytochemical quality. Phytochemical quality advantage zones are more spatially extensive and continuous than fragmented high-suitability areas, suggesting that quality-related spatial signals operate more broadly at the regional scale. This may be because broad-scale environmental variables cannot capture local habitat differences that influence phytochemical accumulation. Bioactive compound accumulation is therefore likely influenced by the combined effects of climate, microhabitat heterogeneity, genetic differentiation, and developmental processes. Incorporating finer-scale ecological variables may further improve the accuracy of habitat suitability and phytochemical quality extrapolation.

In this study, *A. guttata* shows evidence of ecological niche conservatism. Phylogenetic evidence indicates that the genus *Arnebia* originated during the Oligocene and diversified extensively in the middle Miocene, coinciding with global warming events and the progressive uplift of the Tianshan and Himalayan ranges. Long-term evolutionary adaptation and genetic diversity may contribute to this ecological stability (Sun et al., 2022). Intrinsic adaptive traits further reinforce this historically conditioned niche stability. Dense and rigid trichomes reduce transpiration and enhance water-use efficiency under arid and high-radiation conditions (Konrad et al., 2015). Experimental studies also demonstrate strong recovery following drought stress, accompanied by metabolic adjustments that enhance tolerance (Liu et al., 2024b). These traits may buffer environmental variability. As a result, large niche shifts may not be necessary (Wiens and Graham, 2005), which is consistent with the stability of suitable habitats projected by ecological modeling.

Unlike conventional species distribution studies based mainly on climatic variables or single algorithms, this study combined ensemble modeling, phytochemical information, and landscape pattern analysis to examine habitat characteristics of *A. guttata* from multiple perspectives. The spatial distribution of climatically suitable habitats did not fully coincide with phytochemical quality advantage zones, showing that areas favorable for species persistence are not necessarily associated with higher medicinal quality. In addition, landscape fragmentation altered the spatial structure and connectivity of priority habitats after land-use constraints were considered. These results suggest that conservation and cultivation planning for medicinal plants should consider not only climatic suitability, but also medicinal quality and habitat continuity at regional scales.

### 4.2 Environmental determinants and implications for regional cultivation

Understanding the drivers of spatial distribution is improtant for predicting future conservation and cultivation strategies for *A. guttata*. Habitat suitability emerges from the combined influence of climatic, topographic, soil, and anthropogenic conditions. Among these, hydrological variables emerge as the dominant determinants. Such niche requirements are characteristic of arid and semi-arid ecosystems, where water availability constrains plant establishment, phenology, and long-term population persistence more strongly than temperature alone (Currier and Sala, 2022).

Precipitation-related variables largely constrained the broad distribution range of *A. guttata*, whereas soil properties and temperature conditions contributed more to habitat differentiation within suitable regions. Core stable habitats occurred under relatively narrow precipitation ranges and lower climatic fluctuation, while dynamically expanding habitats covered broader hydrothermal gradients. Compared with climatically stable regions, newly suitable habitats under future scenarios may therefore experience greater environmental variability. From a practical perspective, high-quality stable habitats may provide more reliable conditions for future cultivation and experimental planting.

Although the human footprint had relatively weak effects on habitat suitability at the regional scale, human activities may still influence local habitat conditions indirectly. *A. guttata* mainly occurs in remote rocky slopes and desert areas where current disturbance levels remain relatively low. However, infrastructure expansion, grazing, and land-use change can alter habitat connectivity and local microclimatic conditions, which may further increase ecological pressure under ongoing climate change (Feng et al., 2024; Gómez-Márquez, 2025; Guo et al., 2024).

Climate change is expected to continuously reshape the spatial distribution of suitable habitats for wild medicinal plants. However, the emergence of newly suitable areas does not necessarily lead to successful population establishment. Due to limited natural dispersal capacity, many native species are unable to track shifting climatic niches and achieve population renewal in newly suitable habitats without human assistance (Canturk et al., 2026). Therefore, proactive conservation measures, including habitat protection, population reinforcement, and assisted cultivation, are essential for ensuring the long-term persistence of medicinal plant resources under future climate change. Nevertheless, conservation interventions such as grazing exclusion and harvesting control in protected areas may facilitate ecological restoration and improve habitat stability. Our long-term field investigations further indicate that the management of protected areas in China has become increasingly stringent, thereby playing a positive role in promoting the long-term survival of threatened medicinal species. These findings suggest that although human disturbance currently exerts relatively weak direct effects at the regional scale, targeted conservation management remains important for maintaining long-term habitat stability and population persistence.

### 4.3 Ethnomedicinal implications of species

Medicinal plants provide cultural and economic benefits but remain highly sensitive to environmental changes. Wild-harvested species, rooted in traditional knowledge, support rural livelihoods but face growing threats from habitat degradation, knowledge erosion, and unregulated harvesting (Mbelebele et al., 2024). Arnebiae Radix is widely used in traditional medicine in China and Mongolia, though botanical sources differ. In China, it mainly comes from *A. euchroma* (Royle) Johnst. and *A. guttata*, while in Mongolia, *A. guttata* and *A. fimbriata* Maxim. are principal sources. *A. guttata* serves as the shared species utilized in both pharmacopoeial traditions. These ethnobotanical divergences reflect the interplay between resource availability, cultural inheritance, and ecological adaptation. Future expansion along the China-Mongolia border may sustain this region as an important area for both biodiversity and traditional medicinal use. Strengthening collaborative management of *A. guttata* germplasm and habitat resources between the two countries would hold significant ecological and social value. Collaborative conservation actions between China and Mongolia, given the species’ transboundary range and the shared challenges in managing its habitats under changing land-use and environmental conditions.

In recent years, China and Mongolia’s numerous initiatives in the fields of science, technology, and healthcare have strengthened cooperation between the two countries. Given the transboundary distribution of *A. guttata*, collaborative conservation and germplasm management between China and Mongolia will be important for maintaining both biodiversity and traditional medicinal resources under future environmental change. Cross-border cooperation between China and Mongolia has facilitated knowledge exchange, practice standardization, and joint research on the sustainable utilization of traditional medicinal plants.

### 4.4 Research limitations and future directions

Although this study provides valuable insights into the ecological requirements and future distribution dynamics of *A. guttata*, several limitations should be acknowledged. Species distribution models are mainly based on occurrence records and environmental predictors and therefore may not fully capture biological interactions, local adaptation, or evolutionary responses of *A. guttata* under future environmental change (Javeed et al., 2024). Although the ensemble model showed high predictive accuracy, uncertainty still exists in spatial extrapolation, especially in ecological transition zones and environmentally heterogeneous regions. Incorporating long-term field observations, species interactions, and soil microbial processes may help improve the ecological realism of future models. In addition, integrating ecological modeling with population genetics or molecular marker analyses could provide further insight into environmental adaptation and genetic diversity patterns (Veloz et al., 2012).

The relatively small number of phytochemical sampling sites may have limited the predictive performance of the PLSR model and increased uncertainty in the spatial extrapolation of phytochemical quality patterns. Although clear correlations were observed among several structurally related compounds, relationships between environmental variables and phytochemical traits remained comparatively weak. This inconsistency may partly reflect differences in habitat conditions, plant developmental stages, and local population characteristics among sampling sites, as well as the limited sample size available for analysis. In addition, broad-scale environmental variables may not adequately capture the fine-scale ecological conditions associated with secondary metabolite accumulation.

Future studies combining controlled cultivation experiments with field investigations may help separate environmental influences from intrinsic metabolic regulation. Integrating genetic, transcriptomic, and metabolomic approaches could also improve understanding of how environmental factors influence the biosynthesis and accumulation of key bioactive compounds in *A. guttata*. Environmental conditions can also affect the accumulation and translocation of chemical constituents within plants, with marked differences among species and tissues, highlighting the complexity of plant responses to environmental variation (Sevik et al., 2026). Previous studies have shown that multi-omics analyses can help clarify the relationships among gene expression, metabolic pathways, and phytochemical variation under different environmental conditions (Yan et al., 2024). Metabolomic approaches may also provide additional insight into environmentally induced variation in medicinal quality that cannot be fully explained by climatic or soil variables alone (Pant et al., 2021).

Our group is currently conducting seed germination and drought stress experiments to better understand the adaptive capacity and physiological thresholds of *A. guttata* under varying environmental conditions. Future cultivation trials in high-quality stable habitats may help optimize domestication and reduce harvesting pressure on wild populations. At the same time, conservation of wild populations remains essential. Long-term monitoring and adaptive management strategies will be important for evaluating conservation effectiveness and supporting the sustainable utilization of this vulnerable medicinal species.

## 5 Conclusions

This study integrated ensemble species distribution modeling, phytochemical assessment, and landscape analysis to evaluate the future habitat dynamics of the vulnerable medicinal plant *A. guttata* across East Asia. Climatically suitable habitats of *A. guttata* exhibited fragmented and spatially dispersed patterns, whereas phytochemical quality advantage zones were comparatively more spatially concentrated and environmentally differentiated. Despite this heterogeneity, the species largely retained its current ecological niche characteristics across future climate scenarios, reflecting strong niche conservatism within arid and semi-arid ecosystems. Expansion toward the Altai, Qilian, Helan, and Tianshan Mountains may provide additional suitable habitats under future climate conditions, with potential implications for both ecological conservation and medicinal resource development. Environmental controls on distribution shifts and phytochemical quality varied among climatic, topographic, soil, and anthropogenic variables. The accumulation of bioactive compounds appeared partially independent of habitat suitability, suggesting that medicinal quality may also be shaped by intrinsic metabolic regulation and local environmental heterogeneity. These results show that climatically suitable habitats do not necessarily coincide with areas of high medicinal quality or long-term habitat stability. By combining species distribution modeling with phytochemical and landscape analyses, this study identified priority habitats from both ecological and medicinal-resource perspectives. Core stable suitable habitats may therefore serve as important areas for germplasm preservation and future cultivation planning, while high-quality stable habitats deserve additional attention for ecological restoration and sustainable utilization. Future conservation and cultivation strategies should prioritize climatically stable and landscape-connected habitats while integrating medicinal quality assessments into regional resource management and restoration planning.

## Data Availability

The original contributions presented in the study are included in the article/**Supplementary Material**. Further inquiries can be directed to the corresponding authors.
