# Supplementary material for: Integrating habitat suitability, phytochemical quality, and landscape stability to identify conservation priorities for Arnebia guttata Bunge under climate change in East Asia
Source: Front Plant Sci. 2026 Aug 6;17:1890224. doi: 10.3389/fpls.2026.1890224 (PMC13490166; doi:10.3389/fpls.2026.1890224)
Supplement: Supplementary file 1 [file Supplementaryfile1.docx]

Supplementary Material

## 1.1 Supplementary Tables

**Table S1.** The environmental variables used in this study

| No. | Variable | Predictor variables Abbreviations | Unit |
| --- | --- | --- | --- |
| 1 | BIO1 | Annual Mean Temperature | ℃ |
| 2 | BIO2 | Mean Diurnal Range | ℃ |
| 3 | BIO3 | Isothermality (BIO02/BIO07) ×100 | - |
| 4 | BIO4 | Temperature Seasonality (SD ×100) | - |
| 5 | BIO5 | Max Temperature of Warmest Month | ℃ |
| 6 | BIO6 | Min Temperature of Coldest Month | ℃ |
| 7 | BIO7 | Temperature Annual Range (BIO05-BIO06) | ℃ |
| 8 | BIO8 | Mean Temperature of Wettest Quarter | ℃ |
| 9 | BIO9 | Mean Temperature of Driest Quarter | ℃ |
| 10 | BIO10 | Mean Temperature of Warmest Quarter | ℃ |
| 11 | BIO11 | Mean Temperature of Coldest Quarter | ℃ |
| 12 | BIO12 | Annual Precipitation | mm |
| 13 | BIO13 | Precipitation of Wettest Month | mm |
| 14 | BIO14 | Precipitation of Driest Month | mm |
| 15 | BIO15 | Precipitation Seasonality (Coefficient of Variation) | - |
| 16 | BIO16 | Precipitation of Wettest Quarter | mm |
| 17 | BIO17 | Precipitation of Driest Quarter | mm |
| 18 | BIO18 | Precipitation of Warmest Quarter | mm |
| 19 | BIO19 | Precipitation of Coldest Quarter | mm |
| 20 | ASP | Aspect | - |
| 21 | ELE | Elevation | m |
| 22 | SLO | Slope | ° |
| 23 | ALT | Altitude | m |
| 24 | D2_COARSE | Coarse fragments | % |
| 25 | D2_SAND | Sand | % |
| 26 | D2_SILT | Silt | % |
| 27 | D2_CLAY | Clay | % |
| 28 | D2_BULK | Bulk density | g/cm^-3^ |
| 29 | D2_ORG_CARBON | Organic carbon content | % weight |
| 30 | D2_PH_WATER | pH in water | ‘-log(H+) |
| 31 | D2_TOTAL_N | Total nitrogen content | g/kg |
| 32 | D2_CN_RATIO | Carbon nitrogen ratio | - |
| 33 | D2_CEC_SOIL | CEC_soil_ | cmol_c_/kg |
| 34 | D2_CEC_CLAY | CEC_clay_ | cmolc/kg |
| 35 | D2_CEC_EFF | ECEC | cmolc/kg |
| 36 | D2_TEB | TEB | cmolc/kg |
| 37 | D2_BSAT | Base Saturation | % |
| 38 | D2_ALUM_SAT | Aluminum saturation | % |
| 39 | D2_ESP | ESP | % |
| 40 | D2_TCARBON_EQ | Topsoil Calcium carbonate | % |
| 41 | D2_GYPSUM | Gypsum content | % |
| 42 | D2_ELEC_COND | Electric conductivity | dS/m |
| 43 | HF | Human foot | - |

**Table S2.** Land use of the core stable suitable zones

| Basic classification system | Fine classification system | Proportional distribution (%) |
| --- | --- | --- |
| Cropland | Herbaceous rainfed cropland | 11.15 |
|  | Tree or shrub covered rainfed cropland |  |
|  | Irrigated cropland |  |
| Forest | elosed evergreen broadleaved forest | 0.41 |
|  | Closed deciduous broadleaved forest |  |
|  | Open deciduous broadleaved forest |  |
|  | Closed evergreen needleleaved forest |  |
|  | Open evergreen needleleaved forest |  |
|  | Closed deciduous needleleaved forest |  |
|  | Open deciduous needleleaved forest |  |
|  | Closed mixed-leaf forest |  |
|  | forestOpen mixed-leaf forest |  |
| Shrubland | Evergreen shrubland | 1.74 |
|  | Deciduous shrubland |  |
| Grassland | Grassland | 20.58 |
| Tundra | Lichens and mosses | 0.64 |
| Wetland | Swamp | 0.91 |
|  | Marsh |  |
|  | Lake/river flat |  |
|  | Saline |  |
| Impervlous surfaces | Urban impervious surfaces | 1.03 |
|  | Rural impervious surfaces |  |
| Bare areas | Bare areas | 62.85 |
| Water | Water | 0.68 |
| Permanent ice and snow | Permanent ice and snow | 0.01 |

**Table S3.** **Descriptive statistics of eight bioactive compounds in *A.*** ***guttata*.**

| No. | Caffeic acid | Rosmarinic acid | Lithospermic acid B | (-)-Shikonin | Deoxyshikonin | Acetylshikonin | *β,β-*Dimethylacryloylalkannin | Isobutyrylshikonin |
| --- | --- | --- | --- | --- | --- | --- | --- | --- |
| Y1 | 0.0512 | 2.9846 | 5.2561 | 2.8104 | 1.2234 | 0.1909 | 0.5959 | 1.3184 |
| Y2 | 0.0956 | 0.3126 | 0.0543 | 0.2944 | 0.5091 | 0.1560 | 0.1571 | 0.5091 |
| Y3 | 0.0293 | 0.8537 | 0.1361 | 0.8039 | 0.5046 | 0.1097 | 0.1566 | 0.9594 |
| Y4 | 0.0712 | 0.6707 | 0.8045 | 0.6316 | 8.9617 | 3.8398 | 1.4891 | 4.6196 |
| Y5 | 0.1276 | 3.8860 | 2.4304 | 3.6591 | 2.9663 | 1.4546 | 0.4235 | 1.4834 |
| Y6 | 0.0277 | 0.7328 | 0.2909 | 0.6900 | 1.3411 | 0.3743 | 0.3696 | 1.0152 |
| Y7 | 0.0639 | 1.4986 | 2.6390 | 1.4111 | 6.6429 | 2.2743 | 2.0640 | 2.6109 |
| Y8 | 0.0189 | 0.4460 | 0.3924 | 0.4200 | 2.9926 | 0.8447 | 0.2866 | 1.4727 |
| Y9 | 0.4884 | 0.0372 | 0.0141 | 0.0351 | 0.1142 | 0.1481 | 0.1871 | 0.4274 |
| Y10 | 0.0053 | 0.4143 | 0.1072 | 0.3901 | 1.2200 | 0.1742 | 0.6681 | 0.9022 |
| Y11 | 0.1050 | 0.4517 | 0.4721 | 0.4253 | 4.6420 | 0.9046 | 0.3303 | 2.1683 |
| Y12 | 0.0000 | 0.1272 | 0.0794 | 0.1197 | 0.8321 | 0.1930 | 0.3662 | 0.4646 |
| Y13 | 0.0149 | 1.0714 | 1.4941 | 1.0088 | 0.1909 | 0.0557 | 0.1467 | 0.3833 |
| Y14 | 0.0157 | 0.5422 | 0.2578 | 1.0241 | 4.8956 | 0.6160 | 0.5695 | 4.3987 |
| Y15 | 0.0279 | 2.1073 | 0.0881 | 0.1289 | 0.4119 | 0.1379 | 0.1611 | 0.4750 |
| Y16 | 0.0232 | 1.7324 | 0.0170 | 0.0396 | 0.1138 | 0.0613 | 0.1423 | 0.3375 |
| Y17 | 0.0028 | 0.3139 | 0.1017 | 0.2979 | 4.8962 | 1.3041 | 1.0928 | 2.7605 |
| Y18 | 0.0184 | 0.9483 | 0.4512 | 4.6001 | 7.4719 | 3.3728 | 0.9562 | 1.9631 |
| Y19 | 0.0282 | 0.7987 | 0.0368 | 1.0330 | 4.4260 | 1.8701 | 0.4942 | 2.1157 |
| Y20 | 0.0652 | 0.2785 | 0.1064 | 0.2622 | 0.1880 | 0.0846 | 0.0274 | 0.7620 |
| Y21 | 0.0282 | 0.8647 | 0.4853 | 0.7945 | 4.8635 | 2.1544 | 0.5642 | 1.2090 |
| Y22 | 0.1192 | 0.3269 | 0.3255 | 0.3078 | 0.6403 | 0.1513 | 0.2544 | 0.7313 |
| Y23 | 0.0503 | 0.0678 | 0.0966 | 0.0639 | 3.5716 | 0.8422 | 0.4582 | 2.9916 |
| Y24 | 0.0357 | 0.5491 | 1.4510 | 0.5170 | 2.0079 | 0.5712 | 0.5166 | 1.9156 |
| Y25 | 0.0554 | 0.0946 | 0.4512 | 0.0891 | 0.3045 | 0.1167 | 0.0000 | 0.4334 |
| SD | 0.0956 | 0.9325 | 1.1871 | 1.1507 | 2.5854 | 1.0646 | 0.4736 | 1.1988 |
| MEAN | 0.0654 | 0.8844 | 0.7216 | 0.8743 | 2.6373 | 0.8801 | 0.5199 | 1.5371 |
| CV | 146.2136 | 105.4369 | 164.5215 | 131.6089 | 98.0319 | 120.9660 | 91.0855 | 77.9903 |
